# Disruption of Glucose Metabolism in Aged *Octodon degus*: A Sporadic Model of Alzheimer's Disease

**DOI:** 10.3389/fnint.2021.733007

**Published:** 2021-10-11

**Authors:** Pedro Cisternas, Camila Gherardelli, Paulina Salazar, Nibaldo C. Inestrosa

**Affiliations:** ^1^Instituto de Ciencias de la Salud, Universidad de O'Higgins, Rancagua, Chile; ^2^Departamento de Biología Celular y Molecular, Facultad de Ciencias Biológicas, Centro de Envejecimiento y Regeneración (CARE-UC), Pontificia Universidad Católica de Chile, Santiago, Chile; ^3^Centro de Excelencia en Biomedicina de Magallanes (CEBIMA), Universidad de Magallanes, Punta Arenas, Chile

**Keywords:** *Octodon degus*, Alzheimer's disease, aging, glucose, metabolism

## Abstract

Alzheimer's disease is a progressive neurodegenerative disorder and the most common cause of dementia. Although transgenic Alzheimer's disease (AD) animal models have greatly contributed to our understanding of the disease, therapies tested in these animals have resulted in a high rate of failure in preclinical trials for AD. A promising model is *Octodon degus* (degu), a Chilean rodent that spontaneously develops AD-like neuropathology. Previous studies have reported that, during aging, degus exhibit a progressive decline in cognitive function, reduced neuroinflammation, and concomitant increases in the number and size of amyloid β (Aβ) plaques in several brain regions. Importantly, in humans and several AD models, a correlation has been shown between brain dysfunction and neuronal glucose utilization impairment, a critical aspect considering the high-energy demand of the brain. However, whether degus develop alterations in glucose metabolism remains unknown. In the present work, we measured several markers of glucose metabolism, namely, glucose uptake, ATP production, and glycolysis and pentose phosphate pathway (PPP) flux, in hippocampal slices from degus of different ages. We found a significant decrease in hippocampal glucose metabolism in aged degus, caused mainly by a drop in glucose uptake, which in turn, reduced ATP synthesis. Moreover, we observed a negative correlation between age and PPP flux. Together, our data further support the use of degus as a model for studying the neuropathology involved in sporadic AD-like pathology and as a potentially valuable tool in the search for effective treatments against the disease.

## Introduction

Alzheimer's disease is a progressive neurodegenerative disease that culminates with the loss of cognitive functions (Serrano-Pozo et al., 2011; Dubois et al., 2016). Pathologically, Alzheimer's disease (AD) is characterized by the presence of (Aβ)-containing extracellular deposits and intracellular aggregations of hyperphosphorylated tau in the brains of affected patients (Deture and Dickson, 2019; Long and Holtzman, 2019). In addition to protein aggregation, the appearance of clinical symptoms in AD has been associated with a severe reduction in cerebral glucose metabolism, which has been shown to be altered years before cognitive alterations (Small et al., 2000; De Leon et al., 2001; Mosconi et al., 2008b). As the major energy source in the brain, glucose plays a vital role in fueling key neuronal processes, such as synaptic activity maintenance and neurotransmitter synthesis (Harris et al., 2012; Cisternas and Inestrosa, 2017; Dienel, 2019). To ensure proper function and meet its high energetic requirements, the brain requires a continuous supply of glucose, a task that is mainly performed by glucose transporters (Gluts) (Duelli and Kuschinsky, 2001; Shah et al., 2012). Interestingly, a reduction in Glut expression has been observed in the brains of patients with AD and in transgenic mouse models of AD, which exacerbates the pathology by increasing Aβ load and tau phosphorylation, accelerating cognitive impairment and promoting neuronal dysfunction (Liu et al., 2008; Shah et al., 2012; Winkler et al., 2015). Importantly, strategies for restoring glucose have been shown to be protective against Aβ toxicity (Niccoli et al., 2016; Cisternas et al., 2019).

Several rodent models have been used to study AD, among them is *Octodon degus* (degu), a South American rodent with potential in AD research since it naturally recapitulates hallmark pathological features of AD without the need for genetic engineering (Inestrosa et al., 2015; Cisternas et al., 2018). Among these features is the presence of Aβ and tau aggregates, an altered Aβ_42_/Aβ_40_ ratio, astrogliosis, and cognitive decline in aged degus (Inestrosa et al., 2005; Rivera et al., 2016b). Due to the presence of pathological features and the lack of engineered disease-associated mutations, this model has been described to develop sporadic AD (the most common form), which makes this model better at recapitulating the molecular pathways leading to AD. However, whether degus exhibit cerebral glucose alterations similar to those in AD remains unknown. To this end, we tested several markers of glucose metabolism in young (1 and 3 years old) and aged (5 and 6 years old) wild-type captive-born female degus. We found a significant reduction in glucose uptake in the hippocampus and cortex of aged degus, together with a decrease in ATP synthesis and key glycolytic enzymatic activity. Moreover, similar to other AD models, we observed an age-related reduction in the expression of cerebral Gluts (Gejl et al., 2017). Interestingly, the pentose phosphate pathway (PPP) flux was found to be significantly reduced in hippocampal slices from aged degus compared with those from young controls. Together, our results showed severe alterations in brain glucose metabolism in aged degus, further validating it as a natural model of AD that could help identify new therapeutic strategies.

## Materials and Methods

### Animals

Adult female *O. degus* (1, 3, 5, and 6 years old; *n* = 4–5 at each age) were obtained from our colony at the Faculty of Biological Sciences, Pontificia Universidad Católica de Chile. These animals were all derived from wild-caught and captive-born degus. The degus were kept in pairs of related and unrelated females, housed in clear acrylic cages (length × height × depth: 50 cm × 35 cm × 23 cm) with hardwood chip bedding, and provided with water and food (rabbit commercial pellet; Champion, Santiago, Chile) *ad libitum*. Additionally, each cage contained one nest box made of clear acrylic (22 × 12 × 15 cm). The animals were kept in a ventilated room with a natural photoperiod and controlled temperature (yearly minimum = 13.4 ± 0.2°C; yearly maximum = 24.9 ± 0.2°C). All experiments followed the National Institutes of Health guidelines (NIH, Baltimore, MD). All procedures were approved by the Bioethical and Biosafety Committee of the Faculty of Biological Sciences of the Pontificia Universidad Católica de Chile (CBB-121-2013). All efforts were made to minimize the distress and suffering of animals as well as to reduce the number of animals used.

### D-[1-^14^C] Glucose Biodistribution

To study the uptake of glucose in degus *in vivo*, four animals of each age were injected with D-[1-^14^C] glucose *via* the tail vein. Briefly, the degus were anesthetized with isoflurane and injected intravenously *via* the tail with 50 μCi of tracer diluted to a final volume of 20 μl in isotonic saline. Following a 15-min uptake, the animals were killed, and tissues were collected. Tissue radioactivity was quantified by liquid scintillation. D-[1-^14^C] glucose levels were normalized to the weight of the resected tissue and expressed as the percent injected dose (Tsytsarev et al., 2012; Cox et al., 2014). We measured glucose uptake in the whole brain, hippocampus, and cortex as described previously (Cisternas et al., 2019).

### Slice Preparation

Hippocampal slices were prepared as previously described (Cerpa et al., 2010). Briefly, transverse slices (350 μm) from the dorsal hippocampus were sectioned in cold artificial cerebrospinal fluid (ACSF) and incubated for 1 h at room temperature. After incubation, slices were treated to measure glucose metabolism for different lengths of time (0–60 min). The slices were then used in different glucose metabolism assays, namely, glucose uptakes, glycolytic rates, PPP, ATP/ADP levels, the NADPH/NADP+ ratios, and enzyme activity tests, such as HK and G6PDH.

### Quantitative Real-Time PCR (qRT-PCR)

Messenger ribonucleic acid was obtained from hippocampal tissue and used to generate cDNA. Quantitative real-time RT–PCR (qRT–PCR) was conducted using SYBR master mix and 18S mRNA as a control, according to the instructions of the manufacturer, as described previously (Cisternas et al., 2016). As a housekeeping gene, we used cyclophilin, and the values were calculated using the delta Ct and normalized to those of the control gene. Duplicate control reactions for every sample without reverse transcription were included to ensure that the PCR products were not due to amplification of contaminant genomic DNA. We used the following sets of primers: 18S gene, forward 5′-TCAACGAGGAATGCCTAGTAAGC-3′and reverse5′-ACAAAGGGCAGGGACGTAGTC-3′; cyclophilin, forward 5′-TGGAGATGAATCTGTAGGAGGAG-3′ and reverse 5′-TACCACATCCATGCCCTCTAGAA-3′; Glut1, forward 5′-ATGGATCCCAGCAGCAAGAAG-3′ and reverse 5′-AGAGACCAAAGCGTGGTGAG-3′; Glut3, forward 5′-GGATCCCTTG-3′ and reverse 5′-ACCAGTTCCCAATGCACACA-3′; Glut4, forward 5′-CGGCTCTGACGATGGGGAA-3′ and reverse 5′-TTGTGGGATGGAATCCGGTC-3′; hexokinase-1, forward 5′-GGATGGGAACTCTCCCCTG-3′ and reverse 5′-GCATACGTGCTGGACCGATA-3′; phosphofructokinase-1, forward 5′-AGGGCCTTGTCATCATTGGG-3′ and reverse 5′-ACTGCTTCCTGCCTTCCATC-3′.

### Glucose Uptake Analysis

As previously described, the slices were washed with an incubation buffer (15-mM HEPES, 135-mM NaCl, 5-mM KCl, 1.8-mM CaCl_2_, and 0.8-mM MgCl_2_), supplemented with 0.5-mM glucose (Cisternas et al., 2014). Slices were then incubated for 0–60 min with 1–1.2 μCi 2-deoxy-D-[1,2-(N)3H] or glucose [(2-H3)-DG] (PerkinElmer, Waltham, MA, USA) at a final specific activity of 1–3 disintegrations/min/pmol (~1 mCi/mmol). Glucose uptake was arrested by washing the slices with ice-cold PBS, supplemented with 1-mM HgCl_2_. The incorporated radioactivity was quantified by liquid scintillation counting.

### Determination of the Glycolytic Rate

Glycolytic rates were determined as previously described (Herrero-Mendez et al., 2009; Cisternas et al., 2014). Briefly, slices were placed in tubes containing 5-mM glucose and then washed two times in Krebs Henseleit solution, containing the appropriate concentration of glucose. After equilibration in 0.5 ml of Hank's balanced salt solution/glucose at 37°C for 10 min, 0.5 ml of Hank's balanced salt solution containing various concentrations of [3-^3^H] glucose was added, with a final specific activity of 1–3 disintegrations/min/pmol (~1 mCi/mmol). Aliquots of 100 μl were then transferred to another tube, placed inside a capped scintillation vial containing 0.5 ml of water and incubated at 45°C for 48 h. After this vapor-phase equilibration step, the tube was removed from the vial, a scintillation mixture was added, and the ^3^H_2_O content was measured by counting over a 5 min period.

### Measurement of Glucose Oxidation Through the Pentose Phosphate Pathway

Glucose oxidation *via* the PPP was measured as previously described based on the difference in ^14^CO_2_ production from [1-^14^C] glucose (decarboxylated in the 6-phosphogluconate dehydrogenase-catalyzed reaction and in the Krebs cycle) and [6-^14^C] glucose (decarboxylated only in the Krebs cycle) (Cisternas et al., 2014). Slices were washed with ice-cold PBS and collected by trypsinization. The tissue was then resuspended in an O_2_-saturated Krebs Henseleit buffer, and 500 μl of this suspension (~10^6^ cells) was placed in Erlenmeyer flasks with another 0.5 ml of Krebs Henseleit solution containing 0.5 μCi D-[1-^14^C] glucose or 2 μCi D-[6-^14^C] glucose and 5.5-mM D-glucose (final concentration). The Erlenmeyer flasks were equipped with a central well-containing an Eppendorf tube with 500 μl of benzethonium hydroxide. The flasks were flushed with O_2_ for 20 s, sealed with rubber caps, and incubated for 60 min in a 37°C water bath with shaking. The incubations were stopped by the addition of 0.2 ml of 1.75-M HClO_4_ into the main well, and shaking was continued for another 20 min to facilitate the trapping of ^14^CO_2_ by benzethonium hydroxide. Radioactivity was quantified as previously described by liquid scintillation spectrometry (Bolaños et al., 2008; Herrero-Mendez et al., 2009). Both [1-^14^C] glucose and [6-^14^C] glucose were purchased from PerkinElmer (Waltham, MA, USA).

### Quantification of Hexokinase (HK) Activity

Brain slices were washed with PBS, treated with trypsin/EDTA, and centrifuged at 500 × g for 5 min at 4°C. Then, the tissue was resuspended in isolation medium (250-mM sucrose, 20-mM HEPES, 10-mM KCl, 1.5-mM MgCl_2_, 1-mM EDTA, 1-mM DTT, 2-mg/ml aprotinin, 1-mg/ml pepstatin A, and 2-mg/ml leupeptin) at a 1:3 dilution, sonicated at 4°C, and then centrifuged at 1,500 × g for 5 min at 4°C. The HK activity of the supernatant was quantified. For the assay, the purified fraction was mixed with the reaction medium (25-mM Tris-HCl, 1-mM DTT,0.5 mM NADP/Na^+^, 2 mM MgCl_2_, 1 mM ATP, 2-U/ml G6PDH, and 10-mM glucose), and the mixture was incubated at 37°C for 30 min. The reaction was stopped by the addition of 10% trichloroacetic acid (TCA), and the generation of NADPH was measured at 340 nm (Cisternas et al., 2016).

### Quantification of ATP, ADP, and NADPH/NADP^+^

The cellular ADP and ATP levels were measured in slices as previously described using an assay kit according to the instructions of the manufacturer (Cisternas et al., 2019). The NADPH/NADP^+^ ratio was measured using a commercial colorimetric assay, according to the instructions of the manufacturer, as described previously (Sun et al., 2017).

### Determination of Glucose-6-Phosphate Dehydrogenase (G6PDH) Activity

Slices were washed with PBS, collected by trypsinization [0.25% trypsin-0.2% EDTA (w/v)], and pelleted. The tissue was then resuspended in isolation medium (250-mM sucrose, 20-mM HEPES, 10 mM KCl, 1.5 mM MgCl2, 1 mM EDTA, 1-mM DTT, 2 mg/ml aprotinin, 1-mg/ml pepstatin A, and 2 mg/ml leupeptin) at a 1:3 dilution, sonicated at 4°C, and centrifuged for 5 min at 1,500 × g at 4°C. Subsequently, the pellet was discarded, and the supernatant was further separated by centrifugation at 13,000 × *g* for 30 min at 4°C. Finally, the G6PDH activity of the supernatant was quantified in a reaction buffer, containing 1-mM ATP and 10 mM glucose-6-phosphate (G6P) for 30 min at 37°C. The reaction was stopped by the addition of 10% TCA. The generation of NADPH was measured at 340 nm, as described previously (Tsai and Chen, 1998).

### Statistical Analysis

All experiments were performed four to five times (biological replicates), with triplicates of each condition in each experimental run. The results are expressed as the mean ± SEM. The data were analyzed by one-way or two-way analysis of variance (ANOVA) and adjusted for multiple comparisons using Bonferroni's *post-hoc* test; ^*^p ≤ 0.05, ^**^p ≤ 0.01, and ^***^p ≤ 0.001 were considered indicative of significant differences. Statistical analyses were performed using Prism software (GraphPad, La Jolla, CA).

## Results

### Impairment of Cerebral Glucose Uptake in Older Degus

**Figure 1:**
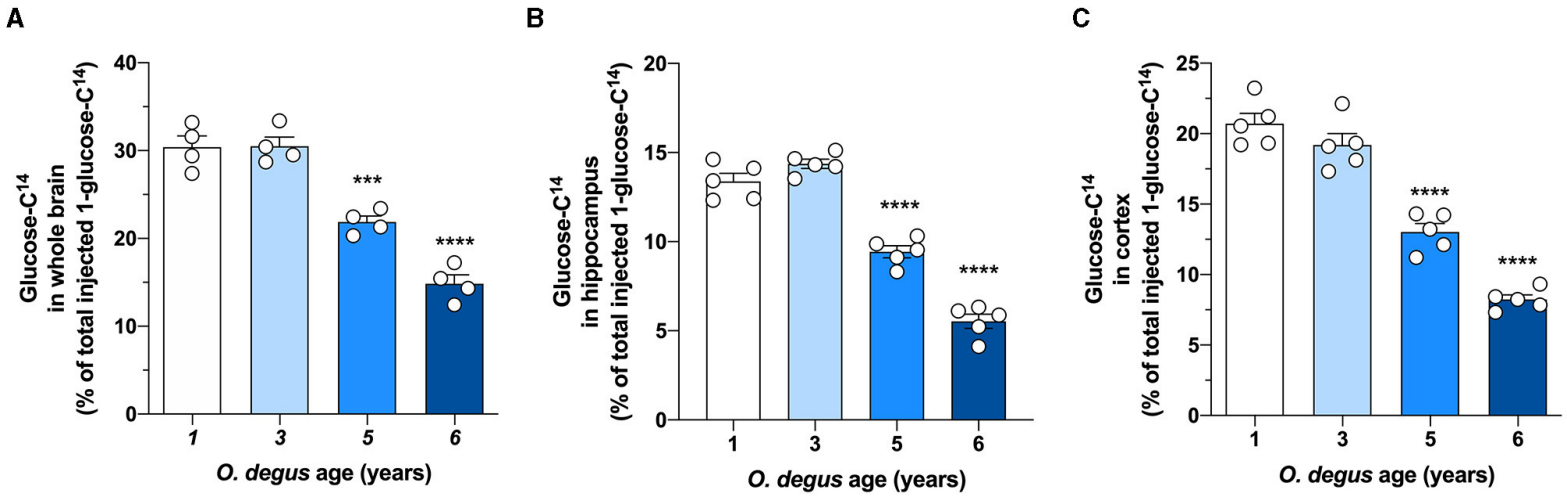
The uptake of radioactive glucose in the whole brain, hippocampus, and cortex is reduced in aged degus. Radioactive glucose was injected *via* the tail vein, and 15 min later, glucose uptake was measured in **(A)** the whole brain, **(B)** hippocampus, and **(C)** cortex in 1- (control), 3-, 5-, and 6-year-old degus. The data represent the mean ± SEM of 4–5 independent biological replicates in three technical replicates. Statistical significance: ***p < 0.001 and ****p < 0.0001, by one-way ANOVA, followed by Bonferroni's *post-hoc* test.

**Figure 2:**
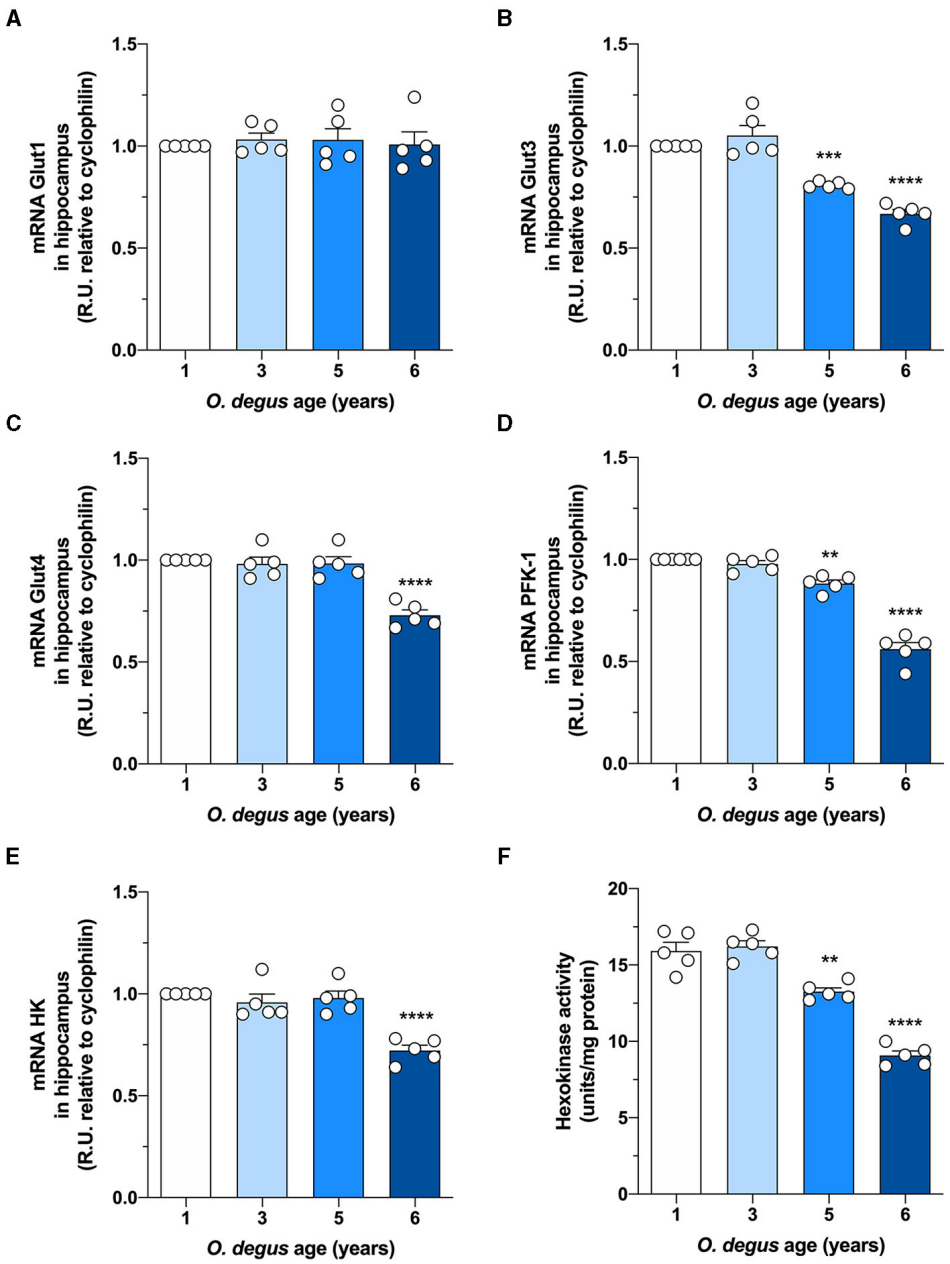
Hippocampal expression of Glut3, Glut4, and glycolytic enzymes is decreased in aged degus. Quantitative RT-PCR of **(A)** Glut1, **(B)** Glut3, **(C)** Glut4, **(D)** phosphofructokinase-1 (PFK-1), and **(E)** hexokinase-1 (HK) expression in hippocampal lysates from 1- (control), 3-, 5-, and 6-year-old degus. **(F)** Hippocampal slices obtained from 1- to 6-year-old degus were used to assess the activity of HK by ELISA. The data represent the mean ± SEM of five independent biological replicates in three technical replicates. Statistical significance: **p < 0.01, ***p < 0.001, and ****p < 0.0001 by one-way ANOVA, followed by Bonferroni's *post-hoc* test.

To evaluate whether degus exhibit alterations in brain glucose metabolism, we first injected animals with D-[^14^C]-glucose (^14^C-G) and followed its uptake in the brain. Our initial analysis showed no differences in whole brain ^14^C-G uptake in 3-year-old animals compared to that in 1-year-old control degus. However, 5-year-old animals exhibited a significant decrease in ^14^C-G uptake, which was further reduced in 6-year-old animals (one-way ANOVA *F*_(3,12)_ = 55.17, *p* < 0.0001, followed by Bonferroni's *post-hoc* test, WT vs. 5 years old, *p* = 0.0004 and WT vs. 6 years old, *p* < 0.0001) (Figure 1A). Importantly, ^14^C-G uptake in the hippocampus and cortex was also observed, with 58 and 60% reductions in 6-year-old degus relative to the control group, respectively (one-way ANOVA *F*_(3,16)_ = 11, *p* < 0.0001 and *F*_(3,16)_ = 79.41, *p* < 0.0001, followed by Bonferroni's *post-hoc* test, WT vs. 6 years old, *p* < 0.0001 and WT vs. 6 years old, *p* < 0.0001 for hippocampus and cortex, respectively) (Figures 1B,C). To evaluate whether the observed disturbances in glucose uptake are caused by alterations in Gluts, we measured the mRNA expression of hippocampal Glut 1, 3, and 4. No differences in Glut1 expression were observed in any of the groups (one-way ANOVA *F*_(3,16)_ = 0.1298, *p* = 0.9410) (Figure 2A). However, Glut3 expression decreased in 5- and 6-year-old degus relative to 1-year-old control animals (One-way ANOVA *F*_(3,16)_ = 43.68, *p* < 0.0001, followed by Bonferroni's *post-hoc* test, WT vs. 5 years old, *p* = 0.0007 and WT vs. 6 years old, *p* < 0.0001) (Figure 2B). The expression of Glut4, on the other hand, showed a significant decrease only in 6-year-old animals compared with 1-year-old animals (one-way ANOVA *F*_(3,16)_ = 23.79, *p* < 0.0001, followed by Bonferroni's *post-hoc* test, WT vs. 6 years old, *p* < 0.0001) (Figure 2C). To gain further insight into glucose metabolism alterations in degus, we measured the expression of two key glycolytic enzymes, phosphofructokinase 1 (PFK1) and hexokinase (HK). HK, the initial enzyme of glycolysis, phosphorylates glucose to glucose 6-phosphate, whereas PFK1 converts fructose 6-phosphate to fructose 1,6-biphosphate. Interestingly, levels of both enzymes were significantly reduced in both 5- and 6-year-old animals for PFK1 and only in 6-year-old animals for HK (one-way ANOVA *F*_(3,17)_ = 110, *p* < 0.0001 and *F*_(3,16)_ = 18.94, *p* < 0.0001, followed by Bonferroni's *post-hoc* test, WT vs. 5 years old, *p* = 0.0022 and WT vs. 6 years old, *p* < 0.0001 for PFK1 and HK, respectively) (Figures 2D,E). Given the reduction in HK expression, we also measured HK activity. Similar to our expression results, HK activity was significantly decreased in 5- and 6-year-old animals relative to controls (one-way ANOVA *F*_(3,16)_ = 72.41, *p* < 0.0001, followed by Bonferroni's *post-hoc* test, WT vs. 5 years old, *p* = 0.0011 and WT vs. 6 years old, *p* < 0.0001) (Figure 2F).

### Glycolytic and Pentose Phosphate Pathway Fluxes Are Reduced in Hippocampal Slices of Older Degus

**Figure 3:**
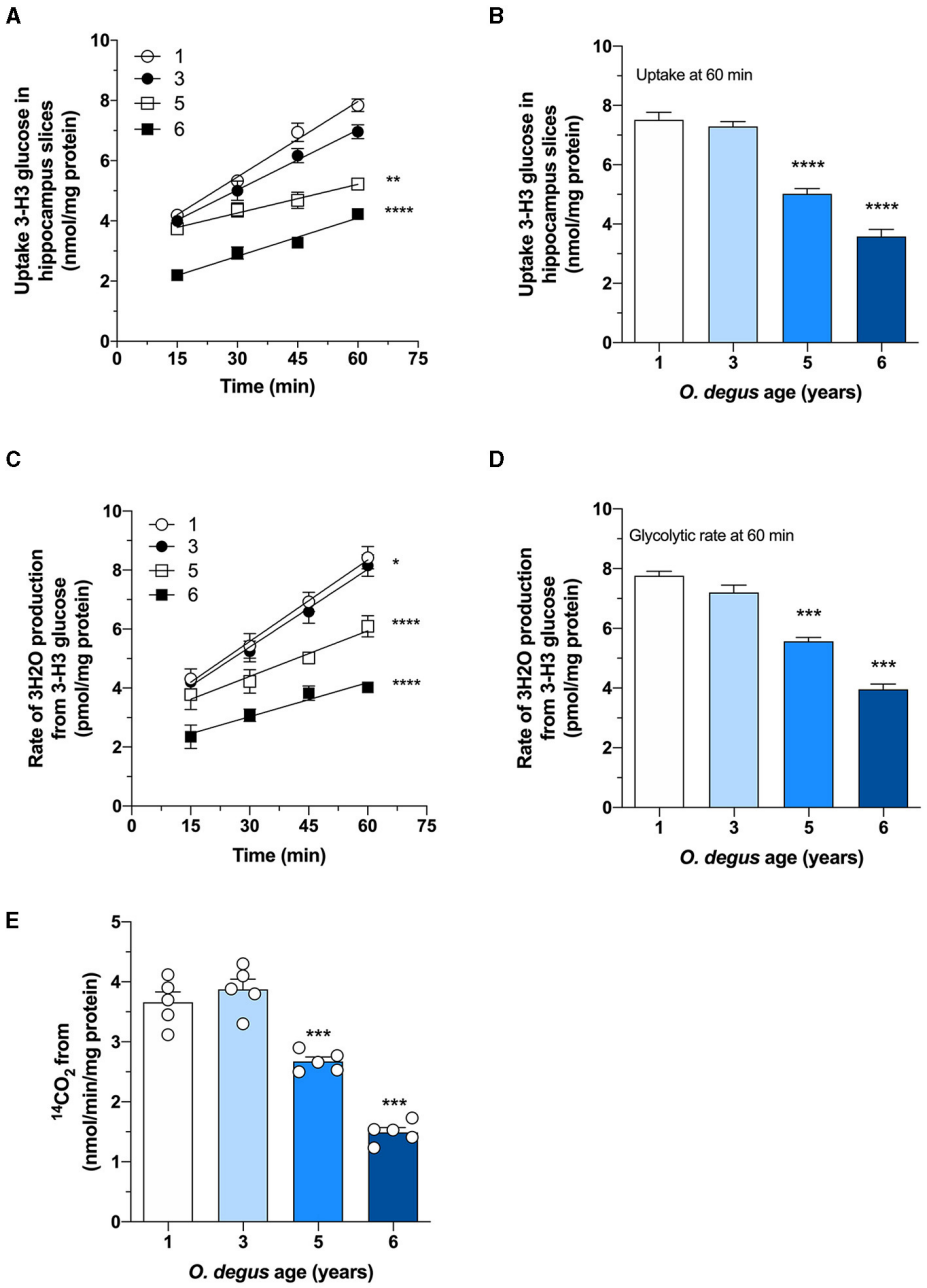
Age-associated reduction in hippocampal glucose metabolism in degus. **(A)** Uptake of radioactive glucose in hippocampal slices obtained from 1- to 6-year-old degus was measured over 60 min. **(B)** Glucose uptake at 60 min of **(A)**. Hippocampal slices from 1-, 3-, 5-, and 6-year-old degus were analyzed for **(C)** glycolytic flux over 60 min. **(D)** Quantification of glycolytic flux at 60 min of **(C)**. **(E)** The pentose phosphate pathway (PPP) was measured in hippocampal lysates of 1- to 6-year-old degus. The data represent the mean ± SEM of five independent biological replicates in three technical replicates. Statistical significance: *p < 0.05, **p < 0.01, ***p < 0.001, and ****p < 0.0001 by one-way ANOVA **(B,D,E)** or two-way ANOVA **(A,C)**, followed by Bonferroni's *post-hoc* test.

**Figure 4:**
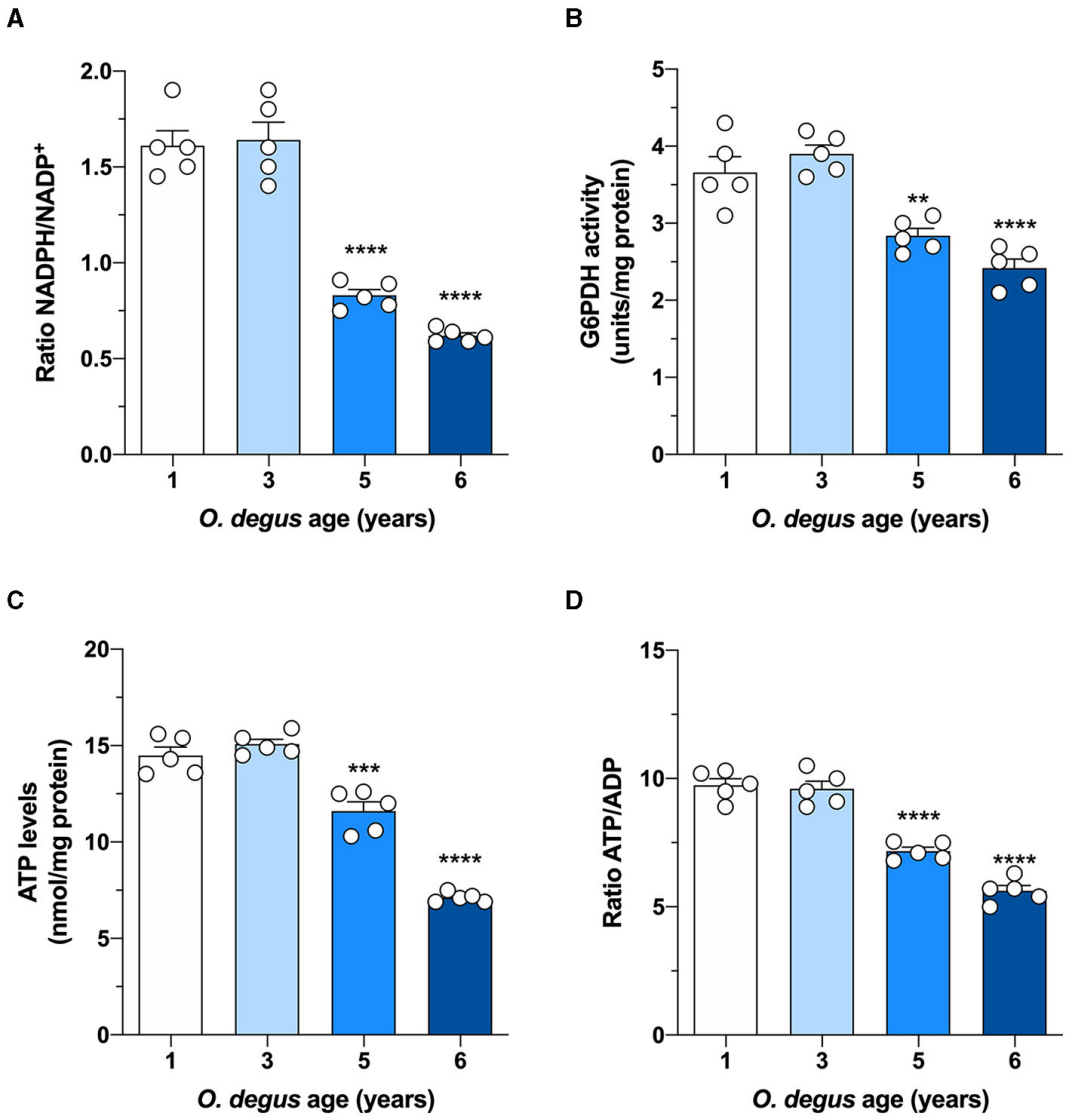
Effect of age on glucose metabolism in degus. Hippocampal slices from 1- to 6-year-old degus were used to assess **(A)** the NADPH/NADP+ ratio, **(B)** glucose-6-phosphate dehydrogenase (G6PDH) activity, **(C)** total ATP, and **(D)** the ATP/ADP ratio. The data represent the mean ± SEM of five independent biological replicates in three technical replicates. Statistical significance: **p < 0.01, ***p < 0.001, and ****p < 0.0001, by one-way ANOVA, followed by Bonferroni's *post-hoc* test.

Once inside the cells, glucose undergoes phosphorylation to form G6P, which can be metabolized by glycolysis to produce ATP and NADH or by the PPP, the main source of NADPH synthesis. To determine whether age can shift G6P utilization in degus, we measured glucose uptake and followed both the PPP and glycolytic fluxes in hippocampal slices. Similar to our *in vivo* results, we found a significant glucose uptake reduction in hippocampal slices from 5- and 6-year-old degus after 60 min compared to those from 1-year-old controls (Two-way ANOVA *F*_(9,38)_ = 4.69, *P* = 0.0003, followed by Bonferroni's *post-hoc* test, WT vs. 6 years old *p* < 0.0001 for Figure 3A. One-way ANOVA *F*_(3,16)_ = 79.71, *p* < 0.0001, followed by Bonferroni's *post-hoc* test, WT vs. 5 years old *p* = 0.0078 and WT vs. 6 years old *p* < 0.0001 for Figure 3B) (Figures 3A,B). Interestingly, the rate of glycolysis was significantly lower in 5-year-old animals than in control animals and further decreased at 6 years; however, no differences were observed in the 3-year-old group (Two-way ANOVA *F*_(9,48)_ = 9.93, *p* < 0.0001, followed by Bonferroni's *post-hoc* test, WT vs. 3 years old, *p* = 0.0253, WT vs. 5 years old, *p* < 0.0001 and WT vs. 6 years old, *p* < 0.0001 for Figure 3C. One-way ANOVA *F*_(3,16)_ = 90.41, *p* < 0.0001, followed by Bonferroni's *post-hoc* test, WT vs. 5 and 6 years old, *p* < 0.0001 for Figure 3D) (Figures 3C,D). Similarly, the rate of glucose oxidized through the PPP in 5- and 6-year-old degus was reduced by ~27 and 59%, respectively, relative to the control (one-way ANOVA *F*_(3,16)_ = 67.05, *p* < 0.0001, followed by Bonferroni's *post-hoc* test, WT vs. 5 years old, *p* = 0.0005 and WT vs. 6 years old, *p* < 0.0001) (Figure 3E). To further confirm the drop in the PPP flux, we also measured the NADPH/NADP^+^ ratio and the activity of glucose-6-phosphate dehydrogenase (G6PDH), the first and rate-limiting enzyme of the PPP. Our results showed an important drop in the NADPH/NADP^+^ ratio of both 5- and 6-year-old animals compared with that of 1-year-old controls (one-way ANOVA *F*_(3,16)_ = 69.86, *p* < 0.0001, followed by Bonferroni's *post-hoc* test, both WT vs. 5 years old and WT vs. 6 years old *p* < 0.0001) (Figure 4A). Moreover, and in accordance with our results, we observed a significant reduction in G6PDH activity in both 5- and 6-year-old degus but not in 3-year-old animals, suggesting an overall decrease in PPP flux (One-way ANOVA *F*_(3,16)_ = 25.06, *p* < 0.0001, followed by Bonferroni's *post-hoc* test, WT vs. 5 years old, *p* = 0.0035 and WT vs. 6 years old, *p* < 0.0001) (Figure 4B). Finally, to determine whether the decrease in glucose uptake has an effect on ATP synthesis, we quantified ATP levels in hippocampal slices. As expected, we observed a significant 20 and 50.8% reduction in ATP levels in 5- and 6-year-old animals compared with those in 1-year-old controls, respectively (one-way ANOVA *F*_(3,16)_ = 105.6, *p* < 0.0001, followed by Bonferroni's *post-hoc* test, WT vs. 5 years old, *p* = 0.0002 and WT vs. 6 years old, *p* < 0.0001) (Figure 4C). To further investigate whether the drop in ATP levels is caused by a reduction in ATP release or by a decrease in ATP production, we also measured ADP levels and calculated the ATP/ADP ratio accordingly. Interestingly, we found a significant reduction in both 5- and 6-year-old animals relative to controls, suggesting a reduction in ATP synthesis (one-way ANOVA *F*_(3,16)_ = 72.69, *p* < 0.0001, followed by Bonferroni's *post-hoc* test, both WT vs. 5 and 6 years old *p* < 0.0001) (Figure 4D).

## Discussion

Cerebral glucose hypometabolism has been used as an early-stage biomarker prior to the onset of clinical symptoms in patients with AD (Mosconi et al., 2008a; Bulleid, 2012; Johnson et al., 2020; Lu et al., 2020; Tams et al., 2021; Zhang et al., 2021). Notably, the stimulation of brain glucose uptake is able to delay AD pathological decline and results in an improvement in cognitive tests, further reinforcing the idea that glucose metabolism in the brain is a fundamental process that is altered in patients with AD (Winkler et al., 2015; Gejl et al., 2017; Lee et al., 2019; Minhas et al., 2021). Among the strategies used to enhance glucose metabolism in the brain, which also result in neuroprotective effects, are the administration of glucagon-like peptide-1, the activation of the Wnt pathway, and antidiabetic agents (Li et al., 2010; Bomfim et al., 2012; Craft et al., 2012; Koenig et al., 2017; Cisternas et al., 2019).

**Figure 5:**
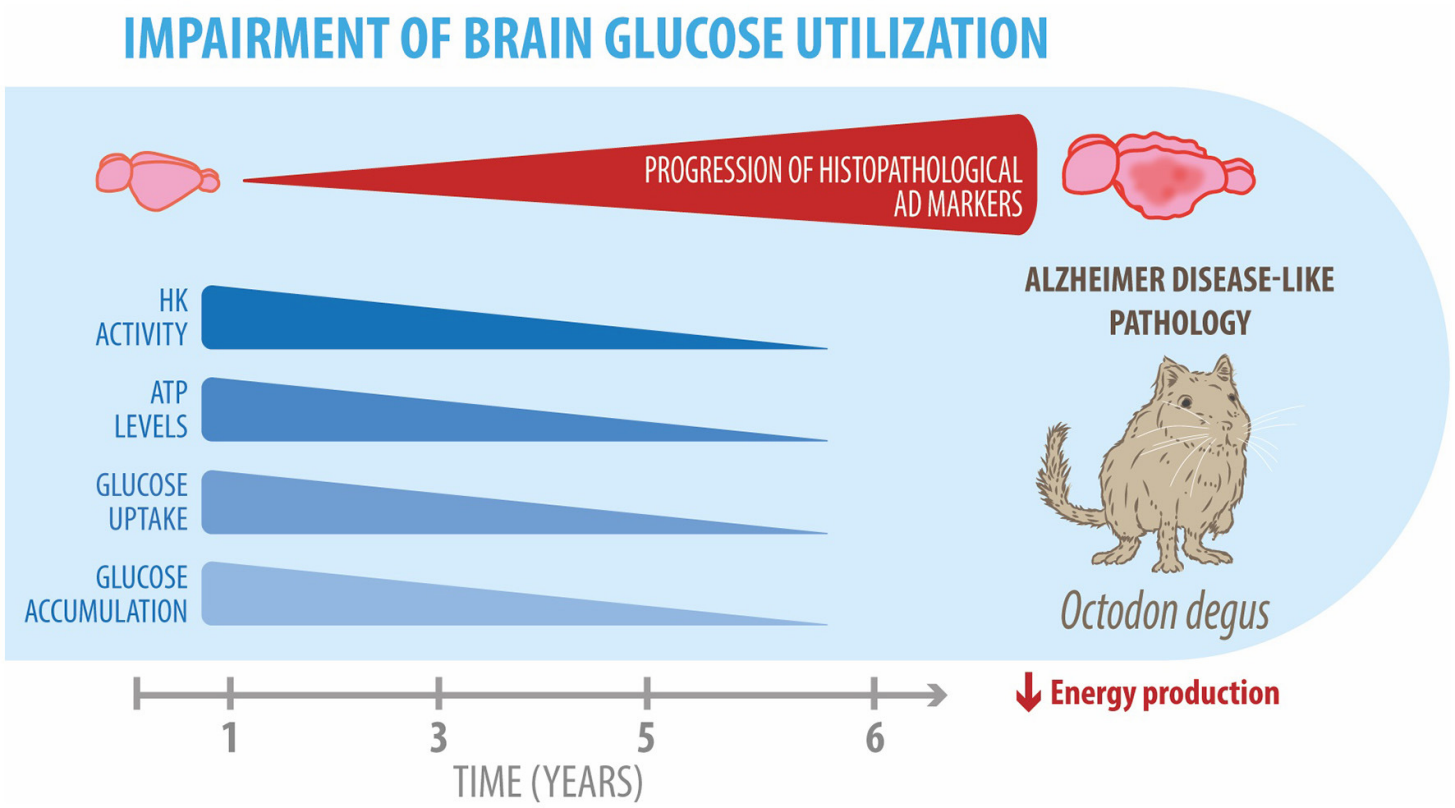
Impairment of brain glucose metabolism in degus. Our data revealed an age-related progressive reduction in several glucose metabolic processes, such as hexokinase expression and activity, ATP levels, and glucose uptake and accumulation in the brains of 5- and 6-year-old degus. Overall, these alterations result in an energetic production deficit, which exacerbates neuroinflammation and neurodegeneration in aged degus.

The degu, a Chilean-endemic rodent, is a promising sporadic model of AD due to the presence of several AD-like neuropathological features. In this study, we evaluated whether degus also develop the cerebral glucose decline seen in patients with AD. Overall, our findings demonstrated glucose uptake decline, Glut expression reduction, and altered key glycolytic enzymes, together with a drop in PPP flux in the brain of aged degus (Figure 5). Our data suggest an age-associated cerebral glucose metabolic decline, further confirming the relevance of *O. degus* as a model for studying AD.

A growing body of literature has demonstrated bioenergetic imbalances in AD (Hoyer, 2004; Mosconi, 2005; Butterfield and Halliwell, 2019). This observation is widely supported by positron emission tomography studies, which have consistently shown a progressive reduction in the glucose metabolic rate in the brains of patients with AD (Mosconi et al., 2009; Ou et al., 2019; Kim et al., 2021). Importantly, these changes in glucose metabolism, possibly caused by abnormal Glut levels and reduced glycolytic flux, have been shown to contribute to the pathogenesis of AD (Ding et al., 2013; Sonntag et al., 2017). Several *in vivo* studies using transgenic mouse models of AD, such as Tg2576 and APP/PS1, have also corroborated a disruption in glucose metabolism (Bigl et al., 2003; Nicholson et al., 2010; Tiwari and Patel, 2014; Waldron et al., 2015; Chen et al., 2021). In accordance with these transgenic models, *O. degus* showed similar glucose metabolism impairments, including a reduction in glucose uptake, together with a decrease in both glycolytic and PPP fluxes.

To enter cells, glucose relies on several facilitative Gluts (Duelli and Kuschinsky, 2001). Numerous Gluts are expressed in the brain, including Glut1, which is highly expressed by endothelial cells in the blood-brain barrier and astrocytes; Glut3, the main neuronal transporter; and Glut4, which is present in several brain areas (Ashrafi et al., 2017; Szablewski, 2017). Our findings showed an age-related decrease in the hippocampal expression of Glut3 and Glut4. These results are consistent with previous data, showing a reduction in the levels of Glut3 and its translocation to the plasma membrane in the brains of patients with AD, as well as in transgenic mouse models of AD (Simpson et al., 1994; Griffith et al., 2019). An age-associated decline in Glut3 in the brains of WT mice was also shown by Ding and coworkers with a further reduction in Glut3 protein levels in AD transgenic mice (Ding et al., 2013). On the other hand, impairments in Glut4 activity have been less consistent in the brain (Steen et al., 2005; Sancheti et al., 2013; Gil-Iturbe et al., 2020). However, a negative correlation between age and Glut4 expression levels has been shown in skeletal muscle (Lin et al., 1991; dos Santos et al., 2012). Interestingly, we observed that the decline in Glut expression occurred earlier for Glut3 than Glut4, at 5 and 6 years old, respectively, suggesting a more central role for Glut3 during aging. Indeed, the lack of neuronal Glut3 in postnatal mice has been shown to shorten the life span, reduce dendritic spines, and diminish cortical thickness, among other alterations, whereas, in adult mice, the absence of Glut3 results in decreased spatial memory and reduced frequency of inhibitory postsynaptic currents (Shin et al., 2018). Notably, several studies have shown a strong and progressive hypoglycemic state in healthy aged individuals (Morrison and Hof, 1997; Cunnane et al., 2011). However, this metabolic decline appears to deteriorate more quickly and abruptly in AD than during normal aging conditions, which could further exacerbate AD pathology (Sims-Robinson et al., 2010; Mosconi, 2013).

We have previously demonstrated that the brains of degus start to age from 3 to 4 years, a stage in which Aβ plaques appear together with neurofibrillary tangles (Inestrosa et al., 2005; Ardiles et al., 2012). These pathological changes also correlate with the appearance of cognitive impairment, a decline in spatial and object recognition memory, and decreased synaptic function (Rivera et al., 2016a). In captivity, however, degus have an increased life span, which could result in more pronounced AD pathology (Steffen et al., 2016). The reason behind this vulnerability has been associated with the sequence similarity of Aβ between humans and degus, with the latter differing by only one amino acid (Inestrosa et al., 2005; Salazar et al., 2016; Steffen et al., 2016; Cisternas et al., 2018). Notably, Aβ sequences from mice and rats differ by only three amino acids from that of humans; however, no transgenic mouse model has been able to recapitulate the neuropathological features exhibited by degus (Castro-Fuentes and Socas-Pérez, 2013; Steffen et al., 2016). It is also worth noting that degus in captivity can be more susceptible to develop diabetes as compared to wild-type animals (Ardiles et al., 2013). Diabetes is a risk factor for developing AD and has been found in people and in several AD models (Kandimalla et al., 2017; de Bem et al., 2021). Indeed, the brains of patients with diabetes show increased deposition of Aβ and hyperphosphorylated tau (Alafuzoff et al., 2009). Moreover, postmortem studies of patients with AD coexisting with diabetes show an increased number of Aβ plaques and other pathological markers when compared with patients with AD or diabetes (Valente et al., 2010). However, the mechanisms that link both disorders remain unclear. Importantly, in our lab, we have monitored glycolysis in degus, and although we have seen metabolic variations, these alterations highly depend on diet and how they are bred (Rivera et al., 2018).

In conclusion, together with previous results from our laboratory, our findings further validate the use of degus as a genetically unmodified research tool to model sporadic AD. The use of degus provides a valuable translational instrument that could help to better comprehend the molecular mechanisms leading to AD and in the development of new therapeutic strategies.

## Data Availability Statement

The original contributions presented in the study are included in the article/supplementary material, further inquiries can be directed to the corresponding authors.

## Ethics Statement

The animal study was reviewed and approved by Bioethical and Biosafety Committee of the Faculty of Biological Sciences of the Pontificia Universidad Católica de Chile.

## Author Contributions

PC and CG: conceived and designed the experiments. PC, PS, and CG: performed the experiments. PC, CG, and NI: analyzed the data. PC and NI: contributed reagents, materials, or analysis tools. CG, PC, PS, and NI: wrote the manuscript. All the authors read and approved the final manuscript.

## Funding

This work was supported by grants from the Basal Center of Excellence in Aging and Regeneration (CONICYT-AFB 170005) and FONIS-Miades T010132 and FONIS-FALZHEIMER T010131 to NI. We also thank the Sociedad Química y Minera de Chile (SQM) for the special grant the role of Lithium in Human Health and Disease.

## Conflict of Interest

The authors declare that the research was conducted in the absence of any commercial or financial relationships that could be construed as a potential conflict of interest.

## Publisher's Note

All claims expressed in this article are solely those of the authors and do not necessarily represent those of their affiliated organizations, or those of the publisher, the editors and the reviewers. Any product that may be evaluated in this article, or claim that may be made by its manufacturer, is not guaranteed or endorsed by the publisher.
